# Advance directives in Finnish long-term care: Do sociodemographic characteristics and regional factors matter?

**DOI:** 10.1177/14034948251356016

**Published:** 2025-07-13

**Authors:** Pirita Forsius, Esa Jämsen, Harriet Finne-Soveri, Mari Aaltonen

**Affiliations:** 1 The Department of Healthcare and Social Welfare, Finnish Institute for Health and Welfare, Helsinki, Finland; 2 Faculty of Medicine, University of Helsinki, Helsinki, Finland; 3 Department of Geriatrics, Helsinki University Hospital, Helsinki, Finland; 4 Faculty of Social Sciences and Gerontology Research Center, Tampere University, Finland

**Keywords:** Advance directives, limitations of treatment, do-not-resuscitate order, end-of-life care, long-term care, RAI assessment, equality in care

## Abstract

**Background:**

Advance directives (AD) help ensure quality end-of-life care by preventing inappropriate or unwanted treatments. This is particularly important for older people in long-term care (LTC).

**Aims:**

This study examines sociodemographic and regional factors associated with the presence of ADs among Finnish round-the-clock LTC residents.

**Methods:**

This retrospective register study included 6,090 Finnish round-the-clock LTC residents aged ⩾65 years who died of chronic progressive diseases in 2019 and had undergone a comprehensive evaluation of health status and care needs using a standardized, internationally accepted tool (interRAI instrument Minimum Data Set 2.0 for LTC).

Regional differences and sociodemographic characteristics associated with the presence of a do-not-resuscitate order (DNR) or other ADs were analyzed using descriptive statistics, chi-squared tests, and multivariable logistic regression.

**Results:**

The most common advance directive was a DNR order (84.8%), while other ADs were less frequent (6.7–22.9%). Both DNR orders and other ADs were associated with female sex, Finnish as the native language, having a legal guardian, and dying of neurodegenerative diseases. The prevalence of DNR orders increased with age. ADs were more common in urban than rural municipalities, but there was substantial variation between municipalities (DNR: 70.9–95.0%, other ADs: 27.1–70.0%). Several associations of sociodemographic and regional factors with ADs remained significant after adjusting for functional ability and health stability.

**Conclusions:**

Sociodemographic characteristics and regional factors influence whether Finnish long-term care residents have advance directives, regardless of their health status. This may result in unequal care and service use despite similar clinical conditions.

## Background

Good end-of-life care respects a person’s wishes and avoids unnecessary and burdensome medical interventions. Nevertheless, many older people receive non-beneficial treatments in their last months of life [1], and burdensome transitions are common [2, 3]. Advance directives (AD) can help in providing quality end-of-life care aligned with a person’s preferences. ADs can refer, for instance, to written care wishes or, more simply, decisions to withhold life-sustaining treatments, such as Do-Not-Resuscitate and Do-Not-Hospitalize orders, nutrition and medication restrictions, and a living will for expressing personal care preferences.

The benefits of ADs are widely recognized. Studies in various countries have found that ADs are associated with improved quality of dying [4], decreased odds of hospitalization [3, 5–7], fewer hospital readmissions [8], less burdensome transitions [9], fewer hospital deaths [10], and decreased use of inappropriate life-sustaining treatments [11]. While the results depend on how care is organized in a given country, providing appropriate care and respect of a person’s right to self-determination are key elements of social and healthcare services everywhere.

ADs are particularly important when a person can no longer express their will, such as in advanced dementia, which affects many older people living in long-term care (LTC). However, the presence of ADs in LTC varies across studies, typically ranging from one-third to two-thirds [5, 12–17], depending on the definition of AD and whether the data are self-reported, staff-reported, or register-based. This variation can also be related to organizational and individual factors.

Earlier studies from European countries, Canada, and the United States suggest that sociodemographic factors may affect the existence of ADs among LTC residents by influencing residents’ willingness to make ADs or care decisions by professionals. ADs appear more common in women [5, 12, 15, 18], widows [12, 15], and older individuals [5, 12, 15, 16, 18]. Regional factors, such as differences between municipalities and urban–rural areas, have been studied less, but divergence seems to exist both between and within countries [12, 15, 16, 19].

Even within Europe, the prevalence of ADs varies substantially between countries [12]. Although some earlier studies are from countries with publicly funded healthcare similar to Finland, they vary in definitions and timing of death and methods used to acquire the data. Some studies even consist of persons outside LTC. The presence of ADs has increased in recent years [14], possibly reflecting changes in guidelines and practices. In fact, Laakkonen et al. [20] found that ADs were mostly explained by LTC characteristics and local care culture.

This study examines the presence of ADs in Finnish round-the-clock LTC and related sociodemographic factors. It also explores regional variation in ADs, which may reflect regional care culture and competence rather than personal characteristics. The study aims to determine which ADs residents had before death, how common they were, and whether sociodemographic characteristics and regional factors were associated with them.

## Methods

### Study design, setting, and ethics

This retrospective register study used individual-level data on Finnish round-the-clock LTC residents who died in 2019 due to chronic diseases. In this study, round-the-clock LTC refers to nursing homes, institutional long-term care, and primary care hospitals providing LTC in Finland.

The data were gathered as part of a national research project entitled “Quality information in palliative care and end-of-life care,” conducted by the Finnish Institute for Health and Welfare. Study approval was obtained through the institute’s internal evaluation procedure (THL/1143/6.02.00/2021 and THL/6000/6.02.00/2021). No approval from an ethics committee or informed consent from the study population was required, as the data for this non-interventional study were retrospectively obtained from national health registers and included only deceased persons.

### Data sources

The information on deaths was collected from the Causes of Death Register (Statistics Finland), which contains data on all deaths in Finland based on official death records. All other data were obtained from the interRAI instrument Minimum Data Set (MDS) 2.0 for LTC, maintained by the Finnish Institute for Health and Welfare, which compiles data from LTC units at the national level. InterRAI instruments are widely used internationally, with different versions available for settings such as home care, LTC, and hospitals, all sharing validated core scales (e.g., cognition, functional ability) alongside setting-specific items. MDS 2.0 is a standardized, comprehensive tool used to assess residents’ health status and care needs in LTC by gathering structured information across a wide range of domains, including medical diagnoses, symptoms, performance in daily activities, psychological factors, social situation, rehabilitation potential, and ADs.

Currently, MDS 2.0 for LTC includes over 300 items and provides data suitable for research, as it contains both individual-level and provider-related information, enabling regional comparisons [21]. In Finland, the MDS assessment is completed by trained nursing staff upon admission to LTC and repeated every six months or when a resident’s condition changes significantly. In 2019, 45% of Finnish LTC residents were assessed using MDS 2.0. Each resident’s most recent MDS assessment completed before the death was used in this study.

### Study population

**Figure 1.**
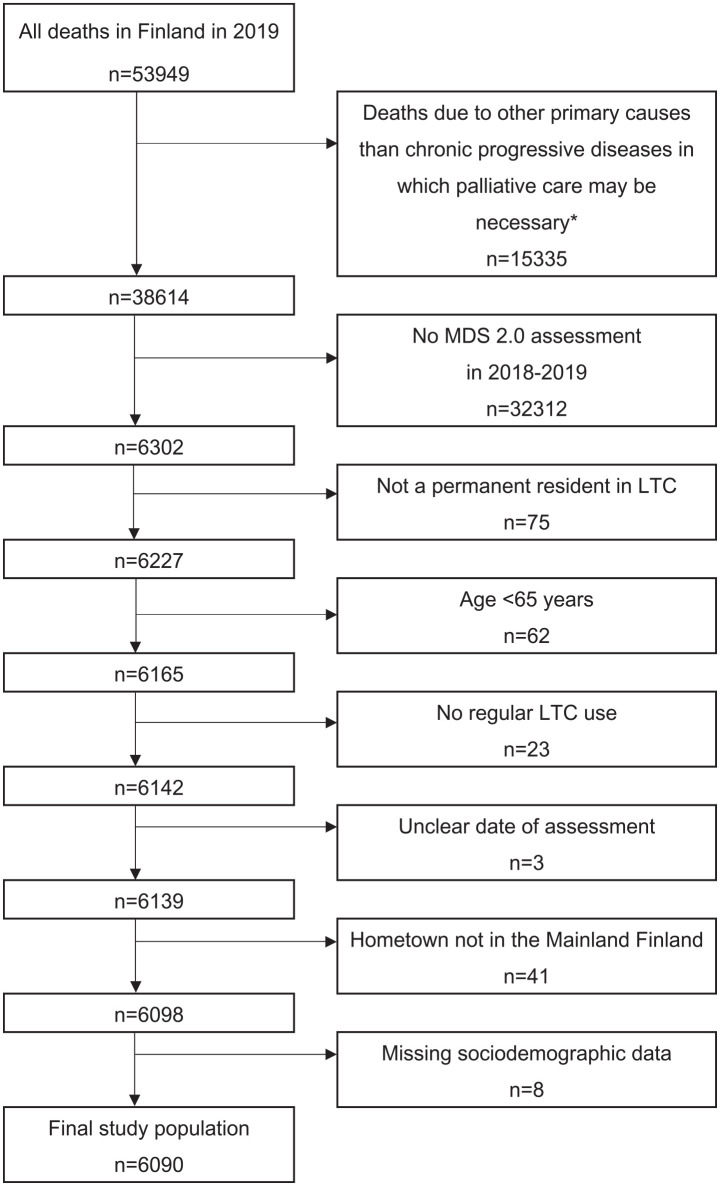
Flowchart presenting the selection of study population. ^a^The codes included were cancers (C00–C97), diabetes (E10–E14), cognitive disorders (F00, F01, F03, G30, R54), central nervous system diseases (G10–13, G20, G23, G30–32, G35–37, G70–73), hypertension-related renal and heart diseases (I11–I14), pulmonary heart disease (I27), cardiomyopathy (I42), heart failure (I50), sequels of cerebrovascular disease (I69), chronic pulmonary diseases (J43, J44, J84, J96.1, J96.9), liver diseases (K70.2, K70.3, K72.1, K72.9, K74), and kidney diseases (N18, N19). MDS: Minimum Data Set; LTC: long-term care.

This study includes Finnish round-the-clock LTC residents who died in 2019 of a chronic progressive disease for which palliative care might be needed at the age of ⩾65 years (Figure 1). These persons were identified from the Causes of Death Register, based on the date of death and the primary cause of death (most important disease underlying the death), registered using the International Classification of Diseases and Related Health Problems 10th Revision (ICD-10).

This sample was linked with MDS assessments using each citizen’s unique personal identification number. Persons with no assessments during the last year of their life (in 2018–2019) were excluded. Persons were also excluded if they were not permanent LTC residents, were <65 years old, or had an unclear assessment date, a hometown outside mainland Finland, or missing sociodemographic data (Figure 1), leaving 6,090 residents for analysis.

### Study variables

#### Resident characteristics

Age, sex, cause of death, and hometown were obtained from the Causes of Death Register. Marital status, language, and guardianship were based on MDS 2.0 assessments. Guardianship refers to a legal arrangement in which another person is appointed to manage the financial and other legal affairs of the resident.

Age was categorized into four groups (65–74, 75–84, 85–94, and ⩾95 years); marital status as unmarried, married or cohabiting, widowed, and separated or divorced; language as Finnish or other (Swedish 8.5%, other languages 0.6%); and guardianship as follows: a legal guardian or other legal oversight, responsible family member or lasting power of attorney, self-responsible resident, and other/unknown. Causes of death were classified as cancer, organ failure, or neurodegenerative disease based on ICD-10 codes.

#### Advance directives

Having an AD was defined as the presence of a living will (LW), do-not-resuscitate order (DNR), do-not-hospitalize order (DNH), feeding restriction, medication restriction, or other care-related restrictions recorded in MDS 2.0.

### Data analysis

Descriptive statistics were used to report the prevalence of any AD and each AD type. Chi-square tests examined sociodemographic differences between LTC residents with and without DNR or other ADs. A logistic regression analysis was executed by entering all sociodemographic variables into the model (Model 1). Since it was assumed that performance in daily activities and cognition affect ADs, the final model (Model 2) was also adjusted for functional ability, cognition, health stability, and time spent in the unit (<1, 1–3, 3–5, or >5 years). MDS items Activities of Daily Living hierarchy (ADLh), Cognitive Performance Scale (CPS), and Changes in Health, End-Stage Disease, and Signs and Symptoms (CHESS) were used to measure functional ability, cognition, and health stability, respectively [22]. The effect of regional differences over the individual characteristics were explored by repeating the analyses for cases from large municipalities with >100 deaths during the observation year (*n*=12). All analyses were performed using SPSS 29.

## Results

The study population included 6,090 residents from 245 LTC units across 180 municipalities. Of these, 3,627 residents (59.6%) were from large municipalities. Approximately two-thirds (65.9%, *n*=4,014) were women, and the median age was 88 years (65–108 years, SD=7.36). The median length of stay in LTC was 886 days (5–7,953 days), and the median time between the MDS assessment and death was 100 days (0–692 days).

In total, 89.4% of the residents had one or more ADs (Table I). The most common AD was a DNR order (present in 84.8%), followed by an LW (in 22.9%). The proportions of other ADs ranged from 6.7% to 8.6%. In the subpopulation from large municipalities, the presence of different ADs varied markedly (Table I).

**Table I.** Presence of advance directives among Finnish round-the-clock LTC residents in the last year before death.

Advance directive (AD)	All municipalities (%)	The municipalities with >100 deaths		
	(*n*=6,090)	(*n*=3,627)		
	Total (%)	Total (%)	Lowest (%)	Highest (%)
Any AD	5,442 (89.4)	3,295 (90.8)	86.2	96.9
Living will (LW)	1,397 (22.9)	963 (26.6)	10.0	63.1
Do-not-resuscitate (DNR)	5,163 (84.8)	3,115 (85.9)	70.9	95.0
Do-not-hospitalize (DNH)	467 (7.7)	304 (8.4)	3.6	26.0
Feeding restriction	444 (7.3)	273 (7.5)	2.9	13.0
Medication restriction	408 (6.7)	231 (6.4)	3.3	19.8
Other restrictions related to care	522 (8.6)	314 (8.7)	4.9	32.1

In bivariate analysis, female sex, Finnish as native language, and living in an urban municipality were associated with a higher likelihood of having a DNR order or other ADs (Table II). ADs were more common in residents dying of a neurodegenerative disease. Advanced age increased the likelihood of having a DNR order (*p*<0.001), but not other ADs (*p*=0.899). Self-responsible residents were less likely to have ADs. Being unmarried decreased the likelihood of having other ADs (*p*<0.001), but not a DNR order (*p*=0.082).

**Table II.** Sociodemographic characteristics of the long-term care residents with a do-not-resuscitate (DNR) order or with other advance directives (ADs) (*n*=6090), based on the interRAI Minimum Data Set 2.0 assessments.

Characteristic	DNR		Other AD		All residents (% of total)
	Present (%)	χ2 / *p*-value	Present (%)	χ2 / *p*-value	
**Age**					
65–74	347 (75.6)	**<0.001**	188 (41.0)	0.899	459 (7.5)
75–84	1,340 (84.0)		631 (39.5)		1,596 (26.2)
85–94	2,762 (85.7)		1,287 (39.9)		3,223 (52.9)
⩾95	714 (87.9)		316 (38.9)		812 (13.3)
**Sex**					
Female	3,442 (85.7)	**0.003**	1,677 (41.8)	**<0.001**	4,014 (65.9)
Male	1,721 (82.9)		745 (35.9)		2,076 (34.1)
**Marital status**					
Widowed	2,790 (85.8)	0.082	1,341 (41.2)	**<0.001**	3,251 (53.4)
Separated or divorced	470 (84.8)		257 (46.4)		554 (9.1)
Unmarried	514 (83.4)		204 (33.1)		616 (10.1)
Married or cohabiting	1,389 (83.2)		620 (37.1)		1,669 (27.4)
**Language**					
Finnish	4,730 (85.5)	**<0.001**	2,235 (40.4)	**0.002**	5,533 (90.9)
Other^a^	433 (77.7)		187 (33.6)		557 (9.1)
**Guardianship**					
Legal guardian or any other legal oversight	1,156 (85.8)	**0.012**	587 (44.1)	**<0.001**	1,347 (22.1)
Family member responsible or a lasting power of attorney	3,888 (84.8)		1,773 (38.7)		4587 (75.3)
Self-responsible resident	21 (70.0)		6 (20.0)		30 (0.5)
Other/unknown	98 (77.8)		56 (44.4)		126 (2.1)
**Cause of death** ^b^					
Neurodegenerative diseases	3,789 (85.6)	**<0.001**	1,813 (41.0)	**0.004**	4,427 (72.7)
Organ failure	1,075 (84.4)		477 (37.4)		1,274 (20.9)
Cancer	299 (76.9)		132 (33.9)		389 (6.4)
**Municipality type**					
Urban	4,064 (85.5)	**0.008**	1,967 (41.4)	**<0.001**	4,752 (78.0)
Semi-urban	540 (82.7)		236 (36.1)		653 (10.7)
Rural	559 (81.6)		219 (32.0)		685 (11.2)

a Other language includes Swedish (8.5%) and foreign languages (0.6%).

b Cause of death: neurodegenerative diseases (F00, F01, F03, G30, R54, G10–G13, G20, G23, G30–32, G35–37, G70–73), organ failure (E10–14, I11–14, I25, I27, I42, I50, I69, J43, J44, J84, J96.1, J96.9, K70.2, K70.3, K72.1, K72.9, K74, N18, N19), cancer (C00–97).

In the multivariable model, increasing age, Finnish as native language, and not having a legal guardian were associated with a higher likelihood of having a DNR order, and female sex and marital status being other than unmarried were associated with a higher likelihood of having other ADs (Table III). Residents in urban areas were more likely to have a DNR order or other ADs than those in semi-urban or rural municipalities. The results related to the cause of death differed between the two models, indicating that functional ability, cognition, health stability, and/or time spent in the unit modify the association. The analysis of the subpopulation from large municipalities showed that the prevalence of DNR orders varied between 71% and 95%, and that of other ADs between 27% and 70% between the communities. The differences remained significant also when differences in resident characteristics were controlled in the multivariable models (Table IV).

**Table III.** Multivariable logistic regression model of sociodemographic characteristics associated with the likelihood of having a do-not-resuscitate (DNR) order or any other advance directive (AD).

		DNR				Any other AD			
		Model 1 OR	95% Cl	Model 2 OR	95% Cl	Model 1 OR	95% Cl	Model 2 OR	95% Cl
Age	65–74	1		1		1		1	
	75–84	1.68*	1.30–2.17	1.69*	1.29–2.21	0.93	0.75–1.15	0.94	0.75–1.17
	85–94	1.89*	1.47–2.42	2.18*	1.67–2.84	0.90	0.73–1.12	0.97	0.78–1.21
	⩾95	2.33*	1.68–3.21	2.78*	1.98–3.90	0.85	0.67–1.10	0.92	0.71–1.19
Sex	Female	1.10	0.94–1.29	0.96	0.84–1.16	1.23*	1.09–1.39	1.14*	1.00–1.28
Marital status	Unmarried	1		1		1		1	
	Married/cohabiting	1.04	0.80–1.36	0.99	0.75–1.31	1.33*	1.09–1.64	1.30*	1.05–1.60
	Widowed	1.07	0.83–1.37	1.11	0.86–1.43	1.48*	1.22–1.79	1.47*	1.21–1.79
	Separated/divorced	1.14	0.83–1.57	1.19	0.85–1.66	1.74*	1.37–2.21	1.72*	1.35–2.20
Language	Finnish	1.66*	1.33–2.06	1.63*	1.29–2.05	1.23*	1.02–1.49	1.20	0.99–1.46
Guardianship	Legal guardian or any other legal oversight	1		1		1		1	
	Family member responsible or a lasting power of attorney	1.77*	1.12–2.79	1.57	0.97–2.54	0.99	0.68–1.44	0.95	0.65–1.39
	Self-responsible resident	1.59*	1.03–2.45	1.47	0.93–2.32	0.81	0.56–1.16	0.79	0.55–1.14
	None of the above/unknown	0.84	0.34–2.08	1.10	0.42–2.92	0.39	0.15–1.02	0.35*	0.13–0.93
Cause of death	Neurodegenerative diseases	1		1		1		1	
	Organ failure	0.94	0.79–1.12	1.44*	1.18–1.75	0.89	0.79–1.02	1.02	0.89–1.18
	Cancer	0.62*	0.48–0.80	1.10	0.83–1.45	0.77*	0.62–0.96	0.93	0.74–1.18
Municipality type	Urban	1		1		1		1	
	Rural	0.80*	0.65–0.99	0.73*	0.58–0.92	0.72*	0.61–0.86	0.68*	0.57–0.82
	Semi-urban	0.82	0.66–1.02	0.77*	0.61–0.97	0.84*	0.71–0.99	0.80*	0.67–0.95

The results are presented as odds ratios (OR) and their 95% confidence intervals (CI). Both models include all listed sociodemographic variables. Model 2 is adjusted also for functional ability, cognition, and health stability (Activities of Daily Living; Cognitive Performance Scale; Changes in Health, End-Stage Disease, and Signs and Symptoms) and time spent in the unit. Statistically significant results are marked with an asterisk.

**Table IV.** Logistic regression model for the subpopulation of the large municipalities (*n*=3,627).

Municipality	DNR					Any other AD				
	*n* (%)	Model 1 OR	95% Cl	Model 2 OR	95% Cl	*n* (%)	Model 1 OR	95% Cl	Model 2 OR	95% Cl
Helsinki	537 (82.4)	1		1		234 (35.9)	1		1	
Espoo	297 (91.4)	2.27*	1.47–3.52	2.55*	1.62–4.01	139 (42.8)	1.34*	1.07–1.75	1.37*	1.03–1.81
Hämeenlinna	144 (70.9)	0.52*	0.36–0.75	0.43*	0.29–0.64	142 (70.0)	4.16*	2.96–5.84	4.17*	2.92–5.93
Jyväskylä	242 (88.0)	1.57*	1.04–2.38	1.36	0.87–2.11	82 (29.8)	0.76	0.56–1.03	0.75	0.55–1.03
Lahti	257 (83.2)	1.06	0.74–1.52	1.12	0.76–1.64	91 (29.4)	0.75*	0.56–0.99	0.80	0.59–1.08
Lappeenranta	136 (92.5)	2.63*	1.38–5.02	2.20*	1.13–4.28	69 (46.9)	1.60*	1.11–2.30	1.71*	1.17–2.49
Oulu	268 (94.0)	3.38*	1.99–5.74	3.20*	1.85–5.55	107 (37.5)	1.07	0.81–1.43	1.08	0.80–1.46
Rovaniemi	123 (93.9)	3.29*	1.57–6.92	3.56*	1.59–8.00	90 (68.7)	3.92*	2.62–5.86	4.19*	2.75–6.37
Tampere	422 (87.6)	1.51*	1.08–2.11	1.41	0.98–2.03	227 (47.1)	1.59*	1.25–2.02	1.66*	1.29–2.14
Turku	320 (78.6)	0.79	0.58–1.08	0.71*	0.51–0.99	222 (54.5)	2.14*	1.67–2.76	2.20*	1.69–2.86
Vaasa	133 (95.0)	4.07*	1.85–8.93	4.54*	2.03–10.15	38 (27.1)	0.67*	0.44–0.99	0.64*	0.42–0.97
Vantaa	237 (87.1)	1.45	0.96–2.18	1.59*	1.034–2.45	114 (41.9)	1.29	0.97–1.72	1.36*	1.01–1.84

The results are presented as odds ratios (OR) and their 95% confidence intervals (CI). Both models include all sociodemographic variables except for the type of municipality. Model 2 is adjusted for sociodemographic characteristics, functional ability, cognition, and health stability (Activities of Daily Living; Cognitive Performance Scale; Changes in Health, End-Stage Disease, and Signs and Symptoms) and time spent in round-the-clock long-term care. Statistically significant results are marked with an asterisk.

## Discussion

This study suggests that sociodemographic characteristics and regional factors influence whether the residents of round-the-clock LTC in Finland have ADs in the last year of their lives. Several sociodemographic characteristics were associated with the presence of ADs, and these associations differed between DNR orders and other ADs. The likelihood of having a DNR or other ADs also depended on the municipality of residence, suggesting that organizational factors and care practices contribute to the presence of ADs, although decision-making should be consistent across similar clinical situations. Hence, Finnish LTC residents may be in an unequal position depending on where they live, even if their functional ability, cognition, and health status are similar.

The high presence of DNR orders suggests that they are a standard care decision in Finnish LTCs, made for most residents. However, there was variation between municipalities in the frequency of DNR orders and even more in other ADs. The presence of other ADs was also very low compared to other studies [5, 13–16], raising the question of whether advance care planning (ACP) has been properly and fully discussed as recommended, since ACP should involve discussing the ADs [23], and ACP interventions are known to increase the completion of ADs [10]. Other ADs besides DNR orders can directly influence a resident’s quality of life and the care they receive at the end of life, while a DNR order is just a limitation of a particular type of care in a specific situation (i.e., cardiac arrest). Therefore, care should be planned comprehensively, considering possibilities for delivering better care.

As in previous studies [5, 15, 16], DNR orders became more common with increasing age. This is reasonable, as diseases often progress and functional ability often deteriorates with age, indicating limited life expectancy. However, in older people, functional ability and need for support can be clearer predictors of prognosis than chronological age [24]. In Finland, access to round-the-clock LTC is possible only with significant functional deficits, and admitted residents usually live the rest of their lives in LTC facilities. Therefore, all LTC residents, regardless of age, should have an ACP and relevant ADs, ideally initiated before LTC admission. Unfortunately, this study shows that this is not the case, supporting an earlier study [25] reporting that, according to emergency physician judgment, most LTC residents that Helicopter Emergency Medical Services encountered should have already had limitations of care in place. Altogether, these observations suggest that more attention must be paid to timely ACP.

Women had an AD more often than men, supporting previous studies [5, 12, 15, 18]. However, in the multivariable model, there was no association with DNR orders, indicating that sociodemographic factors explained the differences. The association remained with other ADs, possibly because women are more often widowed and multimorbid [26], leading to greater consideration of their own wishes. Residents whose native language was not Finnish had fewer ADs. Communicating about limiting treatments in a non-native language can be challenging for both residents and professionals. Despite Swedish being the second official language of Finland, it is possible that Swedish-speaking residents are not having enough discussion about ADs in their own language. Nevertheless, a recent Finnish study found no association between native language and having a living will [18]; therefore, it is possible that a living will is made by the person themselves, without requiring discussions with health care professionals.

Marital status was not associated with having a DNR, but unmarried residents had fewer other ADs than those with other marital statuses. Withdrawing from life-sustaining treatments is more likely when families are involved in end-of-life discussions [27]. Other ADs may reflect personal preferences and interactive decision-making more than DNR decisions. Residents with a legal guardian or responsible family member had ADs more often than those responsible for themselves. This is unsurprising, as many round-the-clock LTC residents have cognitive deficits or advanced dementia, emphasizing the need for early ACP discussions and efforts to listen to people’s wishes before their condition deteriorates and they enter LTC.

A surprising result was that residents dying of cancer were less likely to have an AD. Previously, cancer patients were found to have more DNR orders than those without [28]. In the multivariable analysis, DNR orders were more common in the organ failure group than in the neurodegenerative disease or cancer groups. Those who died of organ failure experience a fluctuating pattern of decline [29], indicating the need for ADs earlier. Patients with organ failure also need hospital care more often, increasing the probability of placing a DNR order. Cancer is often actively treated, but when health and functional ability deteriorate sharply in later stages, ADs may be missed in LTC.

The divergence between rural and urban municipalities may be explained by access to care, use of services, and the organization of medical services in LTC. It could be expected that more attention is paid to ACP if the distance to medical services is long. However, there were fewer ADs in rural and semi-urban municipalities than in urban ones, contradicting Buchanan et al. [19]. Another study found that nursing home residents receiving specialist palliative care were more likely to have an AD [30]. Access to such services may be easier in urban areas.

There were dramatic differences between large, urban municipalities. In 2019, municipalities had organizational and financial responsibility for providing social services such as LTC, leading to variation in organizing services and in the availability of and access to care. Guidelines, recommendations, and competence may also vary between municipalities. These regional differences in how services are organized play a role in the end-of-life process and can lead to LTC residents receiving different care depending on where they live, i.e., inequality in access to and quality of care.

## Strengths and limitations

The strength of this study is inclusion of units from across the country, providing a sufficient and representative sample. Unlike many other studies on LTC, this study looks at the status of ADs in the last year of life when their significance is most central. There were very few exclusions due to missing data, leading to minimal risk of bias. While exclusion of residents aged <65 years or with non-permanent LTC status slightly reduces the generalizability of the findings, it hardly affects the identified associations as the final study population accounted for >96% of all available RAI assessments.

The key limitation is the lack of information in the MDS 2.0 on whether the resident, a proxy, or a physician placed the ADs. This challenges the interpretation of the results, as it is unclear whether the ADs reflect individuals’ preferences or medical decisions. Cultural or religious aspects, which may influence end-of-life decision-making, were not considered. Additionally, there was no information on when the ADs had been placed. Nevertheless, it is noteworthy that many residents close to death lacked them.

Only units using MDS 2.0 were included, potentially leading to a higher presence of ADs in this study compared to the rest of the country, as MDS may direct the staff to pay more attention in placing the ADs. Future research should also explore the role of organizational factors and local practices in ADs.

Lastly, the data are from before the Covid-19 pandemic to avoid confusion with the exceptional situation and temporary practices it brought. The situation with ADs may have changed since the pandemic, as it created a need to review practices and, in some cases, make treatment limitations. Furthermore, during the pandemic, Finland issued national-level instructions to LTC units to update ACP for all LTC residents. However, a recent national report suggested that ACP has not increased in Finnish LTC since the Covid-19 pandemic.

## Conclusions

This study provides new insights into factors associated with having advance directives among round-the-clock long-term care residents in Finland at the end of life. The observed sociodemographic and regional disparities are concerning from an equality perspective. The main purpose of end-of-life care decisions and ADs is to ensure that people receive quality care relevant to their needs, regardless of sociodemographic characteristics or place of residence. Most ADs in long-term care are still DNR orders, and the timeliness of advanced care planning should be improved. As a substantial share of healthcare costs occur in the last year of life, appropriate ADs and early advanced care planning are also relevant from a public health perspective. Regional disparities reflect organizational differences and should be addressed, for example, through education and awareness.
